# Supplementary material for: Distinctive structure of endophytic microbial communities in two species of wild and cultivated rice
Source: Microbiol Spectr. 2025 Jul 23;13(9):e02978-24. doi: 10.1128/spectrum.02978-24 (PMC12403883; doi:10.1128/spectrum.02978-24)
Supplement: Supplemental material — Supplemental figures and tables. [file spectrum.02978-24-s0001.docx]

Figure S1. All sample taxonomic phylogenetic trees


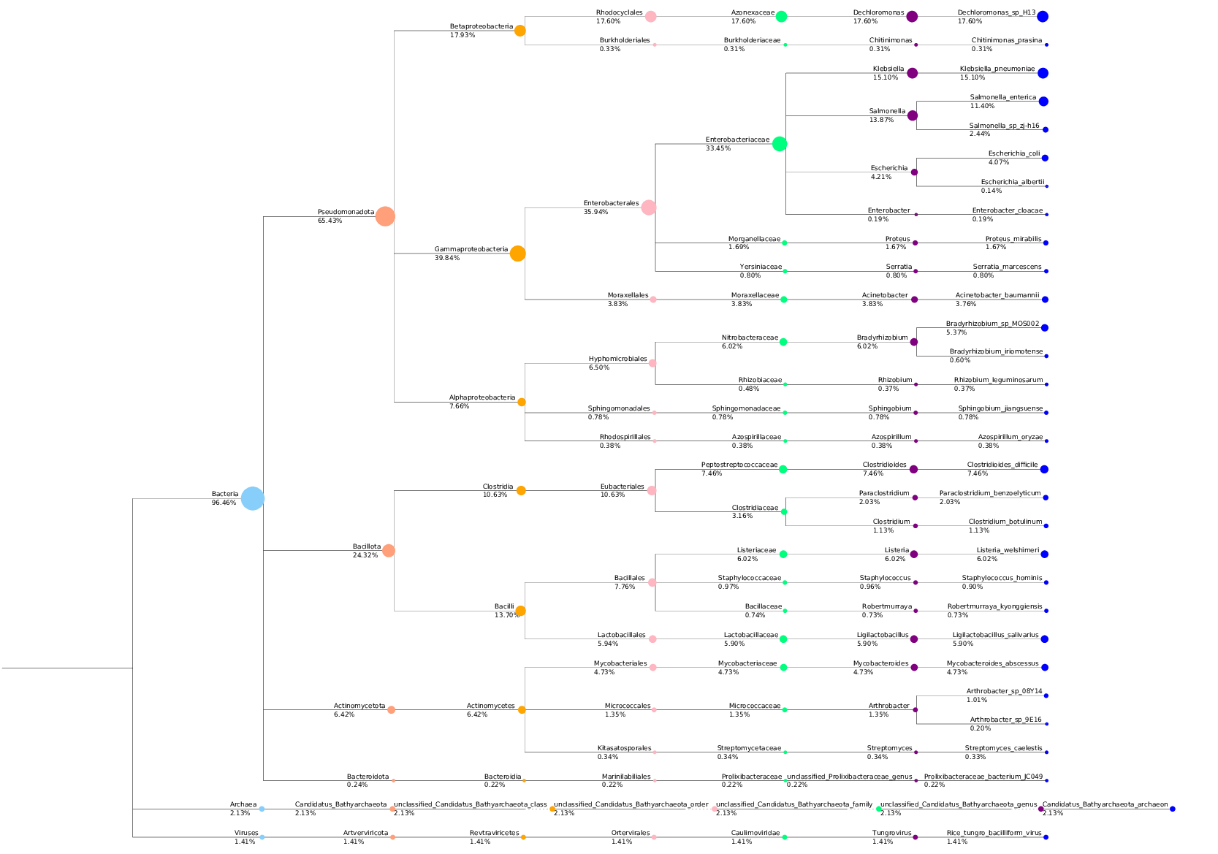


Figure S2. Hierarchical clustering (A) and principal coordinate analysis (PCoA) (B) based on Bray–Curtis distance were conducted to characterize compositional differences in leaf endophytic microbial communities across sample groups.


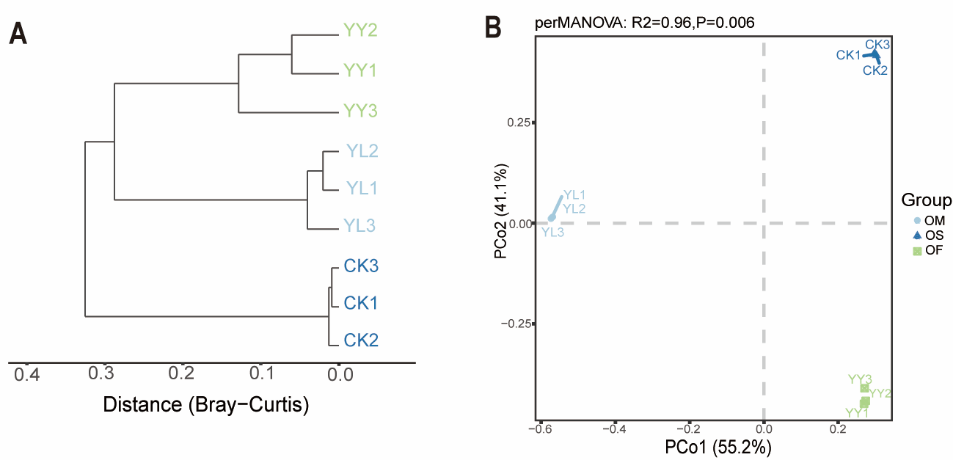


Figure S3. Shared core microbial taxa between wild and cultivated rice.


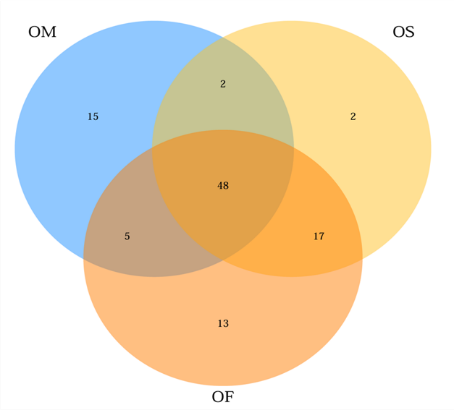


Figure S4. The analysis of the KEGG orthology groups (KOs) of the three groups of samples and the comparisons using a Venn diagram (A). Hierarchical clustering analysis (B) and Principal Coordinates Analysis (PCoA) based on Bray–Curtis distances (C) were performed to dissect the functional differences of leaf endophytes predicted by KEGG among the three sample groups.


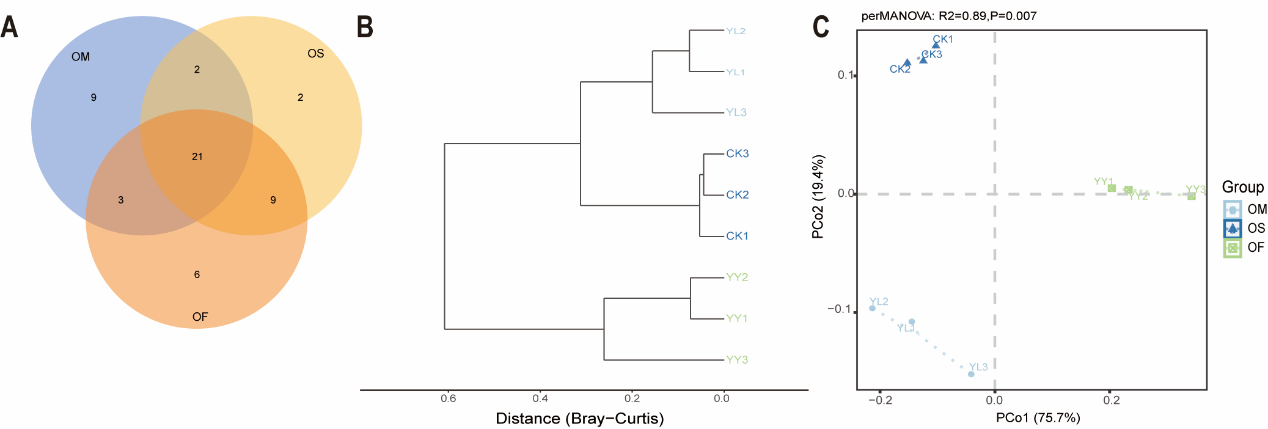


Table S1. Sample sequencing data.

| Sample | Raw reads | Clean reads | Clean reads (%) | Low-quality reads | Q20 base ratio (%) | Q30 base ratio (%) | GC content (%) |
| --- | --- | --- | --- | --- | --- | --- | --- |
| OS 1 | 68834352 | 67973802 | 99.932 | 0 | 98.82 | 96.00 | 45.67 |
| OS 2 | 59843796 | 59125672 | 99.931 | 0 | 98.73 | 95.58 | 45.06 |
| OS 3 | 46554550 | 46019064 | 99.935 | 0 | 98.78 | 95.87 | 45.60 |
| OM 1 | 53588494 | 53101784 | 99.964 | 0 | 98.72 | 95.60 | 47.84 |
| OM 2 | 67387032 | 66801534 | 99.947 | 0 | 98.67 | 95.32 | 47.37 |
| OM 3 | 40935698 | 40534420 | 99.944 | 0 | 98.97 | 96.43 | 49.08 |
| OF 1 | 49668850 | 47866708 | 99.912 | 0 | 99.18 | 97.41 | 47.77 |
| OF 2 | 66504732 | 65541792 | 99.908 | 0 | 98.88 | 96.22 | 45.85 |
| OF 3 | 67857956 | 67221172 | 99.935 | 0 | 98.87 | 96.09 | 45.67 |

Note: OS, Nippon-cultivated rice; OM, *Oryza meyeriana*; OF, *Oryza officinalis*.

Table S2. Composition of microbial communities at the taxonomic level of Kingdom.

|  | Bacteria | Archaea | Viruses | Fungi |
| --- | --- | --- | --- | --- |
| OF | 99.03% | 0.009% | 0.931% | 0.027% |
| OM | 96.52% | 2.132% | 1.411% | 0.002% |
| OS | 99.18% | 0.003% | 0.768% | 0.047% |

Note: OS, Nippon-cultivated rice; OM, *Oryza meyeriana*; OF, *Oryza officinalis*.

Table S3. Relative abundance of shared core microbiota in leaf endophytes of wild and cultivated rice

| Shared core bacterial  species | Relative abundance in OF | Relative abundance in OM | Relative abundance in OS | Corresponding phylum |
| --- | --- | --- | --- | --- |
| *Acinetobacter baumannii* | 8.92 | 3.78 | 5.94 | Pseudomonadota |
| *Aeromonas jandaei* | 0.01 | 0.01 | 0.44 | Pseudomonadota |
| *Algoriphagus aestuarii* | 0.16 | 0.99 | 0.02 | Bacteroidota |
| *Arthrobacter sp. 08Y14* | 0.26 | 0.12 | 0.06 | Actinomycetota |
| *Arthrobacter sp. ZGTC131* | 0.06 | 0.12 | 0.26 | Actinomycetota |
| *Aulographum hederae* | 0.006 | 0.001 | 0.001 | Pseudomonadota |
| *Azospirillum oryzae* | 2.09 | 0.36 | 0.78 | Pseudomonadota |
| *Bacterium LRH843* | 0.008 | 0.002 | 0.004 | unclassified_Bacteria_phylum |
| *Bradyrhizobium iriomotense* | 0.25 | 0.64 | 0.79 | Pseudomonadota |
| *Bradyrhizobium sp. MOS002* | 0.47 | 5.26 | 0.03 | Pseudomonadota |
| *Candidatus athyarchaeota Achaeon* | 0.001 | 2.13 | 0.0002 | Pseudomonadota |
| *Candidatus Symbiopectobacterium* sp. NZEC127 | 0.03 | 0.08 | 0.06 | Pseudomonadota |
| *Chitinimonas_prasina* | 0.09 | 0.34 | 0.99 | Pseudomonadota |
| *Ciceribacter_ferrooxidans* | 0.006 | 0.102 | 0.001 | Pseudomonadota |
| *Clostridioides_difficile* | 9.12 | 7.59 | 0.44 | Bacillota |
| *Clostridium_botulinum* | 0.37 | 1.15 | 1.34 | Clostridium |
| *Dechloromonas sp. H13* | 22.89 | 17.90 | 14.61 | Pseudomonadota |
| *Ectobacillus* sp. SYSU 60031 | 0.020 | 0.009 | 0.030 | Bacillota |
| *Enterobacter cloacae* | 0.48 | 0.18 | 0.49 | Pseudomonadota |
| *Enterococcus faecium* | 0.010 | 0.004 | 0.007 | Bacillota |
| *Escherichia coli* | 5.28 | 4.08 | 2.67 | Pseudomonadota |
| *Flavobacterium* sp. SaA2.13 | 0.026 | 0002 | 0.003 | Bacteroidota |
| *Klebsiella pneumoniae* | 6.32 | 14.76 | 17.90 | Pseudomonadota |
| *Ligilactobacillus salivarius* | 0.31 | 5.80 | 0.02 | Bacillota |
| *Listeria welshimeri* | 17.03 | 6.09 | 4.41 | Bacillota |
| *Luteolibacter flavescens* | 0.07 | 0.03 | 0.05 | Verrucomicrobiota |
| *Mycobacteroides abscessus* | 1.51 | 4.74 | 0.65 | Actinomycetota |
| *Paraclostridium benzoelyticum* | 3.70 | 2.10 | 3.09 | Bacillota |
| *Prolixibacteraceae bacterium* JC049 | 1.24 | 0.23 | 0.25 | Bacteroidota |
| *Proteus mrabilis* | 0.73 | 1.59 | 8.66 | Pseudomonadota |
| *Rhizobium eguminosarum* | 1.67 | 0.37 | 3.38E-05 | Pseudomonadota |
| *Rice ungro acilliform virus* | 0.93 | 1.34 | 0.77 | Artverviricota |
| *Robertmurraya yonggiensis* | 0.47 | 0.73 | 0.01 | Bacillota |
| *Salmonella nterica* | 3.55 | 11.37 | 26.00 | Pseudomonadota |
| *Salmonella* sp. zjh16 | 7.64 | 2.46 | 2.91 | Pseudomonadota |
| *Serratia arcescens* | 0.02 | 0.85 | 0.0002 | Pseudomonadota |
| *Shewanella* sp. A3A | 0.15 | 0.04 | 0.11 | Pseudomonadota |
| *Shewanella* sp. 23 | 0.004 | 0.001 | 0.020 | Pseudomonadota |
| *Sphingobium iangsuense* | 1.40 | 0.80 | 1.23 | Pseudomonadota |
| *Staphylococcus ureus* | 0.03 | 0.05 | 0.02 | Bacillota |
| *Staphylococcus ominis* | 5.78E-05 | 0.913428 | 0.000118 | Bacillota |
| *Streptococcus galactiae* | 0.07 | 0.03 | 0.05 | Streptococcus |
| *Streptomyces lbiflaviniger* | 0.03 | 0.006 | 0.008 | Actinomycetota |
| *Sulfolobus* sp.1 | 0.009 | 0.007 | 0.002 | Crenarchaeota |
| *Unclassified acteria pecies* | 0.006 | 0.002 | 0.309 | Bacteria |
| *Unclassified nterobacteriaceae pecies* | 0.014 | 0.001 | 0.011 | Pseudomonadota |
| *Uncultured acterium* | 0.022 | 0.017 | 0.019 | unclassified_Bacteria_phylum |
| *Vibrio rahaemolyticus* | 0.082 | 0.020 | 0.062 | Pseudomonadota |
